# Identification of Two Novel *EPOR* Gene Variants in Primary Familial Polycythemia: Case Report and Literature Review

**DOI:** 10.3390/genes13101686

**Published:** 2022-09-20

**Authors:** Laura Lo Riso, Gardenia Vargas-Parra, Gemma Navarro, Leonor Arenillas, Lierni Fernández-Ibarrondo, Beatriz Robredo, Carmen Ballester, Bernardo López, Albert Perez-Montaña, Antonia Sampol, Lourdes Florensa, Carles Besses, María Antonia Duran, Beatriz Bellosillo

**Affiliations:** 1 Hematology and Hemotherapy Department, Hospital Universitario Son Espases, IDISBA, 07120 Palma de Mallorca, Spain; 2 Molecular Biology Laboratory, Pathology Department, Hospital del Mar, 08003 Barcelona, Spain; 3 Hospital del Mar Medical Research Institute, IMIM, 08003 Barcelona, Spain; 4 Hematological Cytology Laboratory, Pathology Department, Hospital del Mar, 08003 Barcelona, Spain; 5 Hematology Department, Hospital Punta Europa, 11207 Algeciras, Spain; 6 Hematology Department, Hospital del Mar, 08003 Barcelona, Spain

**Keywords:** erythrocytosis, polycythemia, EPOR, variants

## Abstract

**Simple Summary:**

Erythrocytosis can be caused by a wide variety of diseases. Some forms of erythrocytosis have an obvious cause, such as a kidney injury, or it may have an oncological cause, but in some patients, the origin of the disease is not entirely clear, and since the symptoms of an isolated erythrocytosis are not usually cumbersome, sometimes the diagnosis takes several months or years. In the present work, we report a couple of cases of familial erythrocytosis associated with novel variants in the erythropoietin receptor gene. This study serves as a reminder of the clinical and molecular study of this rare disease and expands the list of mutations associated with primary familial polycythemia.

**Abstract:**

Primary familial and congenital polycythemia is a rare disease characterized by an increase in red cell mass that may be due to pathogenic variants in the EPO receptor (*EPOR*) gene. To date, 33 genetic variants have been reported to be associated. We analyzed the presence of *EPOR* variants in two patients with polycythemia in whom *JAK2* pathogenic variants had been previously discarded. Molecular analysis of the *EPOR* gene was performed by Sanger sequencing of the coding regions and exon/intron boundaries of exon 8. We performed in vitro culture of erythroid progenitor cells. Segregation studies were done whenever possible. The two patients studied showed hypersensitivity to EPO in in vitro cultures. Analysis of the *EPOR* gene unveiled two novel pathogenic variants. Genetic testing of asymptomatic relatives could guarantee surveillance and proper management.

## 1. Introduction

Erythrocytosis is the increase in the number of erythrocytes in peripheral blood. It can be either primary due to an intrinsic defect of the erythroid compartment that is associated with low erythropoietin (EPO) levels or secondary, which is extrinsic to the red cells and associated with normal or high EPO levels. Both primary and secondary erythrocytosis can be acquired or arise from hereditary alterations [1]. Among all types of erythrocytosis, the most common type is polycythemia vera (PV), which is an acquired primary erythrocytosis due to somatic mutations in the Janus kinase gene (*JAK2*). For this reason, apart from excluding secondary causes, the workup of erythrocytosis in the clinical practice includes in the initial testing the screening for *JAK2* mutations [2].

Regarding hereditary alterations causing erythrocytosis, known as primary congenital erythrocytosis (CE), pathogenic variants in the *EPOR* gene have been described in 12-15% of cases, producing defects in the erythropoietin (EPO) receptor (EPOR) [3,4,5]. This entity encompassing alterations in the *EPOR* gene is designated as Primary Familial and Congenital Polycythemia (PFCP) or ECYT1 [6].

The *EPOR* gene codifies for the EPO receptor of 508 amino acids, which belongs to the type I cytokine receptors and consists of an extracellular domain that binds to the EPO ligand, a transmembrane domain and an intracellular domain. Upon EPO binding, the EPOR dimerizes enabling two JAK2 tyrosine kinases, which are pre-attached to the receptor, to become close enough for their transphosphorylation and activation [7]. Activated JAK2 proteins phosphorylate the tyrosine residues located in the intracellular domain of the EPOR. The C-terminal domain contains eight tyrosines that act as binding sites for regulatory proteins such as SHP-1 or SOCS family proteins that dephosphorylate the tyrosine residues of both EPOR and JAK2 [5]. Phosphorylated tyrosine residues become docking sites for other signaling proteins containing SH2 residues, such as STAT5 and PI3K, which are in turn activated by phosphorylation. Activated STAT proteins translocate to the nucleus and activate transcription of genes involved in cell differentiation, division, and apoptosis inhibition [8].

Here, we report two cases of erythrocytosis in which the diagnosis of PV and acquired secondary polycythemia had been ruled out that were further studied for mutations in the *EPOR* gene.

## 2. Materials and Methods

Samples: Two patients presenting sustained erythrocytosis were referred to the Pathology Department of Hospital del Mar. Common causes of secondary erythrocytosis and *JAK2* pathogenic variants in exons 12 and 14 had been previously discarded in both cases. Cases were referred for performing cultures of hematopoietic progenitors and molecular analysis of *EPOR* gene. A total of 20 mL of peripheral blood was collected after informed consent of the patients.

*EPOR* sequencing: The complete coding region and the exon–intron boundaries of *EPOR* gene were sequenced in peripheral blood granulocytes DNA. Sanger sequencing was performed in an ABI3500 instrument after amplification using intronic primers (conditions available upon request). To ensure whether the resulting variants were germinal, variants were confirmed in isolated CD3+ lymphocytes. Variant annotation was performed using the RefSeq accession number NM_000121 for the *EPOR* gene. Segregation studies of *EPOR* variants in family relatives were conducted when possible, using the same *EPOR* sequencing methods.

Next-generation sequencing (NGS): *ASXL1, BCOR, BCORL1, BPGM, CALR, CBL, CEBPA, CHEK2, CSF3R, CSNK1A1, CUX1, DDX41, DLEU7, DNMT3A, EGR1, EGLN1, EPAS1, EPO, EPOR, ETV6, EZH2, FLT3, GATA2, HBA1, HBA2, HBB, IDH1, IDH2, JAK2, KIT, KMT2A, KRAS, MPL, NF1, NPM1, NRAS, PHF6, PPM1D, PRPF8, PTPN11, RAD21, RUNX1, SETBP1, SF3B1, SH2B3, SRSF2, STAG2, THPO, TET2, TNFSF11, TP53, TP53RK, TP53TG5, U2AF1, VHL, WT1*, and *ZRSR2* coding sequences were amplified using two custom GeneRead™ DNAseq Targeted panels (Qiagen). The resulting libraries were sequenced on a NextSeq^®^ platform (Illumina) and analyzed with the QIASeq DNA pipeline (Qiagen). Variants obtained were filtered and annotated with the Illumina VariantStudio software and visualized with the Integrative Genomics Viewer.

Cell culture of hematopoietic progenitors: Endogenous erythroid colony (EEC) assay was conducted and scored precisely as previously described [9]. To assess erythropoietin sensitivity, peripheral blood mononuclear cells from patients and controls were plated in methylcellulose medium (2 × 10^5^/mL; Methocult H-4533, Stem Cell Technology). Epo was added at the following concentrations: 0.0, 0.03, 0.06, 0.125, 0.25, 0.5, 1, 3, and 4 IU/mL. Cultures were maintained in a humidified atmosphere with 5% carbon dioxide at 37 °C. Large erythroid colonies (BFU-E) were scored at day 14.

Literature review and variant classification. We searched for *EPOR* variants using the terms (EPOR)+(variants) or (EPOR)+(mutations) in *PubMed* and selected the variants that were reported to be directly associated with PFCP. We classified the variants following the ACMG/AMP general guidelines [10].

## 3. Results

We have identified two novel *EPOR* pathogenic variants in two cases referred to the Molecular Diagnostics Laboratory in Hospital del Mar (Barcelona).

Case 1 was a 17-year-old woman of Spanish origin with hemoglobin (Hb) levels of 18.6 g/dL and hematocrit of 62%, who had severe headaches. The only family history related was a 32-year-old brother who resides in Germany, who also had been treated with phlebotomies. Case 2 was a 55-year-old male from Syria who presented with Hb levels of 17.4 g/dL and hematocrit of 52.6%, severe iron deficiency, and severe headaches, mental dullness, and asthenia; his father presented a history of phlebotomies. He had three children of 18, 16, and 12 years old with no clinical/analytical alterations.

Both patients had low levels of serum EPO (<1.5 mU/mL) excluding hypoxia-driven polycythemia. At physical examination, no splenomegaly was reported in any of them, leucocyte and platelet counts were normal, and bone marrow biopsies showed no panmyelosis.

Cell culture of hematopoietic progenitors showed a slight endogenous growth of erythroid cell colonies in the absence of EPO in case 1. Hypersensitivity to EPO was observed in both cases.

**Figure 1:**
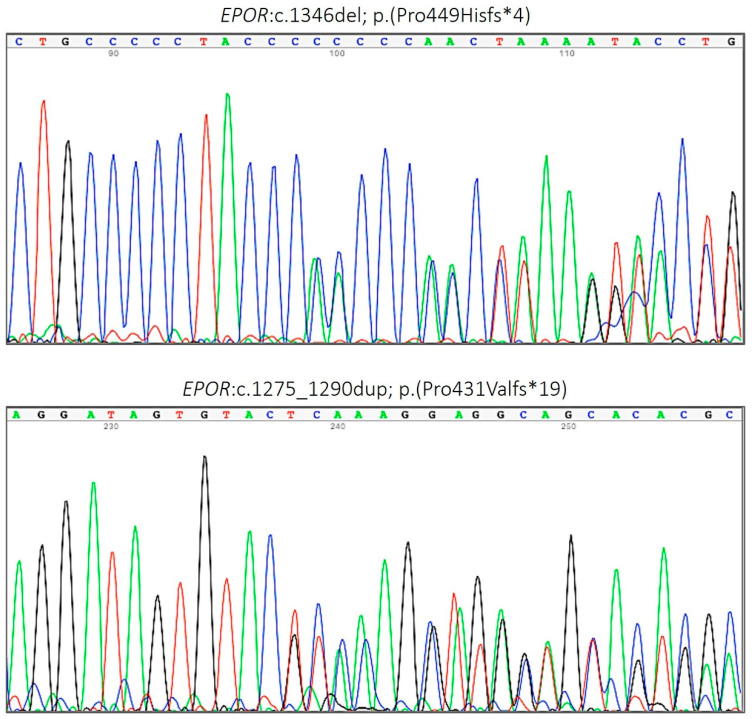
*EPOR* pathogenic variants identified in this study.

Sanger sequencing of the exon 8 of *EPOR* showed the presence of two novel mutations: in the first case, a duplication of 16 nucleotides leading to a frameshift at proline 431 and a subsequent termination codon after 19 amino acids (c.1275_1290dup, p.Pro431Valfs*19) and in the second case, a deletion of 1 cytosine also causing a frameshift, with the substitution of the proline 449 for a histidine and the appearance of a stop codon after four amino acids (c.1346del, p.Pro449Hisfs*4) (Figure 1). The two variants produced a shorter protein with loss of six of the eight tyrosine residues involved in phosphorylation signaling and were therefore considered as pathogenic.

These findings were corroborated in the NGS analyses, and no further pathogenic variants were found in genes related to somatic or germline erythrocytosis.

Segregation studies were attainable in the brother of case 1 and in all three healthy children of case 2. The 32-year-old brother, who also had erythrocytosis and low EPO levels, resulted as a carrier of the same c.1275_1290dup variant. *EPOR* sequencing of case 2′s healthy children revealed that his 18-year-old son was the only of the three inheriting the c.1346del variant.

All 33 *EPOR* variants reported to be associated with PFCP to date, and their classification are listed in Table 1. According to ACMG/AMP guidelines, 26 of them are classified as pathogenic or likely pathogenic, 3 are variants of uncertain significance, and 4 are benign or likely benign.

## 4. Discussion

PFCP is an autosomal dominant disease with incomplete penetrance, characterized by an isolated primary polycythemia associated with increased red cell mass and subnormal EPO levels. It mimics the clinical presentation of PV, a neoplastic (clonal) disorder, whereas in PFCP, the hematopoiesis is polyclonal, benign, and usually shows good response to phlebotomies if needed [34]. It is important to correctly diagnose these patients in order to avoid labeling them as “neoplastic”.

*EPOR* gain of function variants account for around 12% of the ECYT1 suspected patients [4,35]. In our experience, *EPOR* pathogenic variants were found in 6% (2/34) of all studied patients, presenting polycythemia of unknown origin and no *JAK2* pathogenic variants.

Congenital predisposition to erythrocytosis can be caused by mutations in a wide range of genes such as *EPOR*, *VHL*, *EGLN1*, *EPAS1, HBB, HBA1, HBA2*, and *BPGM*. Until 2008, there were 11 *EPOR* pathogenic variants reported as causative of familiar polycythemia. Nevertheless, some of the previously reported variants are classified as benign or likely benign according to the current ACMG/AMP guidelines [10]. In Table 1, we classified all *EPOR* variants reported to date following these criteria. Our cases expand the list of *EPOR* described mutations so far, and we can expect this number to rise soon with the inclusion of *EPOR* in NGS panels [36].

In a small proportion of patients with idiopathic erythrocytosis (IE), the presence of probably pathogenic variants in the *SH2B3* gene has been reported. This gene is also associated with myeloproliferative neoplasms, especially when they are associated with variants in *LNK* or other genes of the JAK2 pathway [37]. Therefore, it would be recommended that in cases with erythrocytosis and normal levels of EPO, all these genes be studied at the same time whenever possible, betting on the use of NGS approaches. In the case of acquired mutations, we would be able to assess clonality and in IE possible multilocus inherited alleles.

Since there is a great number of already-existing variants of uncertain significance (VUS), and more is to come as NGS is more widely used, standardized functional studies are a must. Here, we performed cell culture proliferation assays to demonstrate hypersensitivity of hematopoietic progenitors containing pathogenic variants in *EPOR* to low concentrations of EPO as a demonstration of pathogenicity of these variants. These studies have been previously used as a functional analysis for mutations in this gene [28,38].

Previously reported pathogenic mutations in the *EPOR* gene produce a shorter EPOR that lacks the docking sites for the negative regulators in the EPO signaling [39]. SHP-1 is a protein tyrosine phosphatase mainly expressed in hematopoietic cells, which inactivates EPOR upon binding to a tyrosine residue located at the C-terminal tail [40]; presumably, loss of these residues, due to truncation mutations, results in an EPO receptor lacking a SHP-1 binding site at Y454, maintaining the observed activation of the EPO receptor. The two novel mutations identified here also cause a shorter protein in which six of the eight conserved tyrosine residues, including Y454, are lost. It has been widely reported that truncations of the EPOR that lack all negative regulatory sites are sufficient to produce erythrocytosis [6]. Pasquier et al. demonstrated that frameshift mutations leading to a new C-terminal cytoplasmic tail including a MDTVP motif increase EPO dimerization and stability [28] compared with wild type and even when compared with its nonsense equivalent lacking the MDTVP motif. Case 1, showing a duplication of 16 base pairs, results in the formation of a new cytoplasmic tail containing this MDTVP motif.

## 5. Conclusions

We have reported two new mutations in the *EPOR* gene (c.1275_1290dup and c.1346del). The presence of mutations in *EPOR* establishes the diagnosis of PFCP, an underdiagnosed disease of benign origin that can cause a severe and disabling disease and that, once diagnosed, is usually easy to treat.

## Figures and Tables

**Table 1:** PFCP-associated *EPOR* variants reported to date.

Exon	Nucleotide Change	Protein Effect	Variant Type	References	Class
8	c.1013G>A	p.(Cys338Tyr)	Missense	[11]	VUS
8	c.1022C>T	p.(Thr341Met)	Missense	[11]	VUS
8	c.1138C>G	p.(Pro380Ala)	Missense	[12]	B
8	c.1183G>C	p.(Val395Leu)	Missense	[13]	LB
8	c.1142_1143del	p.(Pro381Glnfs∗2)	Frameshift	[14]	PAT
8	c.1195G>T	p.(Glu399∗)	Nonsense	[15]	PAT
8	c.1220C>A	p.(Ser407∗)	Nonsense	[16]	LPAT
8	c.1228A>C	p.(Gln343Pro)	Missense	[13]	VUS
8	c.1234del	p.(Ser412Argfs∗41)	Frameshift	[17]	PAT
8	c.1235C>A	p.(Ser412∗)	Nonsense	[12,18]	PAT
8	c.1242_1276del	p.(Ser415Hisfs∗18)	Frameshift	[12,18]	PAT
8	c.1249G>T	p.(Glu417∗)	Nonsense	[19]	PAT
8	c.1252 1255del	p.(Gly418Profs∗34)	Frameshift	[20]	PAT
8	c.1271_1272del	p.(Phe424∗)	Nonsense	[14]	PAT
8	c.1273G>T	p.(Glu425∗)	Nonsense	[3]	LPAT
8	c.1275_1290dup	p.(Pro431Valfs*19)	Frameshift	Present study	PAT
8	c.1278C>G	p.(Tyr426∗)	Nonsense	[21,22]	PAT
8	c.1281dup	p.(Ile428Tyrfs∗17)	Frameshift	[23]	PAT
8	c.1283_1289dup	p.(Ser432Glyfs*15)	Frameshift	[24]	PAT
8	c.1285del	p.(Leu429Trpfs∗24)	Frameshift	[14]	PAT
8	c.1288dup	p.(Asp430Glyfs∗15)	Frameshift	[25]	PAT
8	c.1293del	p.(Ser432Alafs∗21)	Frameshift	[26]	PAT
8	c.1299_1305del	p.(Gln434Cysfs∗17)	Frameshift	[15,23]	PAT
8	c.1300C>T	p.(Gln434∗)	Nonsense	[27]	PAT
8	c.1300dup	p.(Gln434Profs*11)	Frameshift	[28]	PAT
8	c.1310G>A	p.(Arg437His)	Missense	[12,18,29]	LB
8	c.1311_1312del	p.(Pro438Metfs∗6)	Frameshift	[30]	PAT
8	c.1316G>A	p.(Trp439∗)	Nonsense	[31,32]	PAT
8	c.1317G>A	p.(Trp439∗)	Nonsense	[22]	PAT
8	c.1346del	p.(Pro449Hisfs∗4)	Frameshift	Present study	PAT
8	c.1362C>G	p.(Tyr454∗)	Nonsense	[29]	LPAT
8	c.1460A>G	p.(Asn487Ser)	Missense	[14,33]	LB
8	c.1462C>T	p.(Pro488Ser)	Missense	[23,25]	LPAT

Abbreviations: B, benign; LB, likely benign; VUS, variant of uncertain significance; LPAT, likely pathogenic; PAT, pathogenic.

## Data Availability

Not applicable.
